# Associated factors to caries experience of children undergoing general anaesthesia and treatment needs characteristics over a 10 year period

**DOI:** 10.1186/s12903-020-01302-4

**Published:** 2020-11-04

**Authors:** Katrin Bekes, Antonia Steuber, Nadia Challakh, Jana Schmidt, Rainer Haak, Valentina Hraský, Dirk Ziebolz

**Affiliations:** 1 Department of Pediatric Dentistry, School of Dentistry, Medical University of Vienna, Vienna, Austria; 2 Department of Preventive Dentistry, Periodontology and Cariology, University Medical Center Goettingen, Goettingen, Germany; 3 Department of Cariology, Endodontology and Periodontology, University of Leipzig, Liebigstr. 12, 04103 Leipzig, Germany

**Keywords:** General anesthesia, Children, Dental care, Caries index, Body mass index

## Abstract

**Background:**

Aim of this study was to describe the characteristics of 1- to 6-year-old children who underwent general anesthesia (GA) in a German specialized pediatric dental institution between 2002 and 2011, and to evaluate the risk factors (age, migration background, nutritional status) for caries experience (dmf-s) in these children.

**Methods:**

A cross-sectional study with retrospective data collection was designed. Children who underwent comprehensive dental treatment under GA were enrolled in the study. The data were collected from patient records and included personal background: age, sex, dmf-s, nutritional status, reasons for GA and treatments provided. Mann–Whitney-U test, Chi-square tests, and linear regression modelling were applied for statistical analyses.

**Results:**

652 children (median age: 3 years [IQR: 2–4], 41.6% female) were treated under GA between 2002 and 2011. Of these, 30.8% had migration background, 17.3% were underweight and 14.8% overweight. The median dmf-s was 28 (IQR: 19–43.5). Univariate, only age and migration showed a significant association with dmf-s (*p* < 0.01) up to the age of 5 years. In the linear regression analysis, this association of dmf-s with age (OR: 4.04/CI: 2.81–5.27; *p* < 0.01) and migration (OR: 4.26/CI: 0.89–7.62; *p* = 0.013) was confirmed. At the patient level, tooth extraction was the most chosen option in both time periods, however, more restorative approaches were taken between 2007 and 2011 including pulp therapy and the use of strip and stainless steel crowns compared to 2002–2006.

**Conclusions:**

Children aged 1–6 years treated under GA showed a high caries experience (dmf-s), whereby age as well as migration, but not BMI, were relevant risk factors. Although tooth extraction is the first choice in most cases in the first time period, more conservative procedures were performed in the second half of the follow-up period.

## Introduction

Dental caries still remains a global public health problem [1], although a substantial decline in prevalence in children in many parts of the world has been observed [2, 3]. This caries decline is characterized by a strong polarization, leaving a considerable group of children with very high levels of dental caries and treatment needs [4]. If left untreated, dental caries can cause pain and infection [5]. Moreover, the disease can affect general health [6] due to its impact on children’s nutrition, growth and development. An association between dental caries and BMI has also been suggested [7]. A BMI outside the ideal range may be a risk factor for dental caries, but may also be considered an outcome of dental caries [8]. Nevertheless, in young children with early childhood caries (ECC) the link between ECC and obesity is controversial [9–12].

While the majority of children with caries can be successfully treated with simple behavior modification techniques such as “tell, show and do” [13] under local analgesia (LA) alone, some children fail to respond and require other modalities for anxiety and pain management for treatment to be successfully delivered [14]. Especially very young children with extensive dental decay as well as highly anxious and children, who are unable to comply with the demand of treatment due to behavioral management problems belong to this group [15]. General anesthesia (GA) is a very efficient treatment modality for these children, as it only takes a single appointment and requires little or no cooperation from the side of the patient [16]. GA results in total relaxation, and recall of the procedure is minimized [17]. However, it is only regarded as the last resort, because general anesthesia may pose risks for the patient’s overall health [16].

The characteristics of comprehensive dental treatment under GA have been reported in several studies [14, 16, 18–20]. However, the literature reveals a lack of scientific studies on the trends in pediatric dental treatment performed under GA over a longer time period and simultaneously analyze risk factors such as nutritional status. To our knowledge, only Chen et al. [21] assessed treatment patterns in Taiwan over such a long observation period. Additionally, Alkilzy et al. [22] compared changes in treatment approaches under GA at two time points.

Therefore, the aim of this retrospective (comparative), monocentric cross-sectional study was two-fold: Firstly, it evaluated possible risk factors such as age, migration background and nutritional status for caries experience (dmf-s) in these children. Secondly, it analyzed the characteristics of treatments of children aged 1–6 years who underwent GA in a German spezialized pediatric dental institution between 2002 and 2011 in different time periods (2002–2006, 2007–2011).

Accordingly, the caries experience (dmf-s) was the primary outcome of this study; two main null hypothesis for this study were formulated: (1) there is no association between caries experience (dmf-s) and BMI index, and (2) there is no difference in terms of characteristics of treatment in both time periods.

## Materials and methods

### Patients

This retrospective database, monocentric cross-sectional study analyzed a cohort of children aged 1–6 years who were treated under GA at the Department of Preventive Dentistry, Periodontology and Cariology, University Medical Centre Goettingen, Germany, between 01.01.2002 and 31.12.2011.

The study was carried out in accordance with the Declaration of Helsinki, and the study protocol was reviewed and approved by the ethics committee of the University Medical Centre of the Georg-August-University, Goettingen, Germany (application no. 2/6/12). All patients and the authorized persons (parents) were informed in verbal and in written form about the scientific use of the clinical data prior to clinical data collection (before dental rehabilitation under GA). Written informed consent was obtained.

Study participants were identified based on the records found in the database of the Department. Inclusion criteria for retrospective data collection and analysis were: children aged 1–6 years and the provision of written informed consent prior to dental rehabilitation; exclusion criteria were incompleteness of dental records and missing written informed consent.

All of the children were seen for a preoperative assessment. At this appointment, administrative and clinical details related to the patient were recorded. Radiographs were performed when possible; if this was not possible, these steps were undertaken at the beginning of the GA. Different pediatric dentists trained at the same institution determined the need for GA and performed all dental procedures. As this study was retrospective and based on patient records, calibration was not carried out before the treatments. The required preventive, endodontic, restorative (fillings, stainless steel crowns), and surgical treatments for each patient were completed in a single session under GA with nasal intubation. Teeth with deep carious lesions displaying pulp exposition received pulpotomy or if possible endodontic treatment (with complete pulpectomy and filled with calcium hydroxide paste), depending on indication. Otherwise, the treatment of choice was extraction.

### Data collection

Data were extracted from patients´ dental records. Information considering age, sex, body weight (measured in kg) and height (measured in cm), general disease (e.g. diabetes mellitus) and migration background = at least one of their parents previously entered their present country of residence as a migrant (yes or no) were obtained. Body mass Index (BMI) was analyzed based upon weight and height. After adjusting for age, nutritional status was classified in: underweight, normal weight and overweight [23].

According to the dental examination, the dmf/s index was obtained for evaluation of caries experience. A tooth/surface was considered as decayed (*d*) if a carious lesion (without consideration of initial carious lesions) was found, as missing (*m*) if the reason for loosening the tooth was caries and filled (*f*), if there was a restoration in the tooth/surface [24].

Furthermore, information considering the reason for dental treatment in GA (e.g. age and no compliance because of anxiety), date of GA appointment, form of anesthesia (general anesthesia with or without preoperative sedation) and duration of GA were obtained. The performed therapeutic measures (e.g. tooth extraction, fillings and stainless steel crowns or strip crowns, professional tooth cleaning) and success in completion of dental therapy (yes or no) were recorded.

### Statistical analysis

All statistical analyses were performed with SPSS Version 24.0 (SPSS Inc., U.S.A.). The data analysed within the present study did not show normal distribution (Shapiro–Wilk test).

Thus, associations of age, sex, nutritional status and migration background with caries experience (dmf-s) were analyzed using Mann–Whitney-U and Chi-Squared test. Considering differences in therapeutic measures between the two periods of time (2002 until 2006 vs 2007 until 2011) chi-squared tests were performed. Differences between groups were regarded as significant for *p* < 0.05. Multivariable linear regression model was used for analyzing the influence of migration background and age (factors which showed statistically significant influence in univariate analysis) on the presence of carious lesions on surface level.

## Results

### Description of the study population

Between 2002 and 2011, 652 children aged 1–6 years (41.6% female) received GA. In general, sex, migration background and nutritional status did not differ significantly between the two treatment periods (2002–2006 and 2007–2011; *p* > 0.05; Table 1). In both time frames, “age” was the most common reason for carrying out GA (62.1% vs 51.4%). Between 2007 and 2011, significantly more patients were treated in GA due to being non-compliant (*p* = 0.03) as well as having general diseases (*p* < 0.001). Considering caries experience, no statistically significant differences were observed between both time periods (*p* = 0.3) (Table 1). For 401 study participants dmf-s as well as age, migration status were available, in 344 children dmf-s and nutritional status (Table 2).

**Table 1:** Description of the study sample for both periods of time

Time frame	Sample size (N = 652)			*p*
	2002–2011 (n = 652)	2002–2006 (n = 334)	2007–2011 (n = 318)	
Age (median, IQR) [years]	3; 2–4	3; 2–4	3; 2–4	**0.031**^†^*
% female (n)	41.6 (269)	43.1 (144)	39.0 (138)	0.24^#^
% migration background (n)	30.8 (201)	27.5 (92)	32.5 (115)	0.06^#^
% general disease (n)	8.7 (57)	4.5 (15)	13.2 (42)	**< 0.001**^#^*
BMI (median, IQR) [kg/m^2^]	n = 556 15.6; 14.6–17.0	n = 276 15.9; 14.6–17.2	n = 280 15.4; 14.5–16.9	
Nutritional status	n = 555	n = 276	n = 279	0.56^#^
% underweight (n)	17.3 (96)	17.4 (48)	17.2 (48)	
% normal weight (n)	67.9 (377)	66.3 (183)	69.5 (194)	
% overweight (n)	14.8 (82)	16.3 (45)	13.3 (37)	
dmf-t (median, IQR)	n = 478 10.0; 7.0–13.0	n = 224 10.0; 7.0–13.0	n = 254 10.0; 7.0–13.0	**0.21**^†^
dmf-s (median, IQR)	n = 401 28.0; 19.0–43.5	n = 162 29.0; 20.8–44.0	n = 239 28.0; 18.0–43.0	**0.30**^†^
Reason for treatment				**0.03**^#^*
% age (n)	61.8 (403)	66.2 (221)	57.2 (182)	
% no compliance (n)	19.8 (129)	16.8 (56)	23.0 (73)	
% treatment need (n)	18.3 (119)	17.1 (57)	19.5 (62)	
% risk of bleeding (n)	0.2 (1)	0 (0)	0.3 (1)	

Bold indicates significance level: *p* < 0.05

*Statistically significant

^†^Mann–Whitney-U test

^#^Chi-squared test

**Table 2:** Median values of dmf-s in dependence of different influencing parameters

Parameter	dmf-s [median; IQR]	n
Nutritional status (n = 344)		
Underweight	30.0; 21.5–48.5	53
Normal weight	28.0; 18.0–42.0	236
Overweight	27.0; 20.0–49.2	55
Age [years]* (n = 401)		
1	19.0; 16.3–28.8	12
2	24.0; 18.0–31.8	108
3	29.0; 16.3–45.8	100
4	32.0; 23.0–48.3	90
5	39.0; 22.8–51.3	62
6	40.0; 26.5–56.5	29
Sex (n = 401)		
Female	30.0; 20.0–45.0	171
Male	27.0; 18.0–42.8	300
Migration* (n = 401)		
No	26.0; 18.0–42.0	260
Yes	34.0; 24.0–48.0	141

*Statistically significant differences between groups; N: number of participants for whom considered information were available; n: number of participants revealing the corresponding parameter

### Influence of age, sex, migration background and nutritional status on caries experience

In univariate analysis neither sex nor nutritional status showed an influence on dmf-s (*p* > 0.05). However, statistically significant associations were found for age (*p* < 0.001) and migration background (*p* = 0.001) and dmf-s. Six year-old patients showed the most caries experience with a median dmf-s of 40 (Table 2). Patients with migration background showed significantly higher dmf-s than those without migration background (Median of 34 vs 26, *p* < 0.001, Table 2) in univariate analysis. With the estimates 4.04 (CI 2.81–5.27; *p* < 0.001) of age and 4.26 (CI 0.89–7.62; *p* = 0.013) of migration background, linear regression analysis confirmed and modelled these parameters’ influence on dmf-s (Table 3). Age and migration background explain 11.5% of variance in dmf-s (corrected R^2^ = 11.5).

**Table 3:** Linear regression model in the age group 1–6 years for dmf-s and influencing factors age and migration background (n = 401 cases included)

	Estimate (CI)	Std. error	p
(Intercept)	16.91 (12.43–21.40)	2.28	< 0.001
Migration	4.26 (0.89–7.62)	1.71	0.013
Age	4.04 (2.81–5.27)	0.63	< 0.001

Corrected R^2^ = 11.5

### Dental treatment

Table 4 shows the treatments being recorded between 2002 and 2011. Significant differences were found for the performance of strip crowns, stainless steel crowns, pulpotomies, extractions and fillings on patient level as well as overall (*p* < 0.05). Significantly more patients were treated with strip crowns, stainless steel crowns, pulpotomies and fillings (different materials) between 2007–2011 compared to 2002–2006 (*p* < 0.001, Table 4). Although tooth extractions were considered first choice in both time periods, these were significantly more often performed in the first time period (*p*_patient_ < 0.001/*p*_overall_ = 0.007, Table 4). Furthermore, the kind of anesthesia differed significantly in the different periods of time with a higher percentage of additional sedation before GA in the years of 2002 until 2006 and significantly more sole GA from 2007 until 2011, respectively (*p* < 0.001, Table 4). Overall, in the years of 2002 until 2006 there were significantly less therapies completed (51.7%) compared to the period of 2007 until 2011 (66.9%, *p* < 0.001, Table 4).

**Table 4:** Therapy in the different time period on patient level

Therapy	Sample size (N = 589)			*p*
	2002–2011 (n = 589)	2002–2006 (n = 330)	2007–2011 (n = 259)	
% strip crown (n)	9.1 (59)	0.9 (3)	17.8 (56)	**< 0.001**^#^*
% stainless steel crown (n)	13.2 (86)	0.9 (3)	26.2 (83)	**< 0.001**^#^*
% endodontic treatment (n)	0.6 (4)	0.3 (1)	0.9 (3)	0.361^#^
% pulpotomy (n)	10.2 (66)	0 (0)	20.8 (66)	**< 0.001**^#^*
% extraction (n)	93.6 (603)	97.0 (321)	90.1 (282)	**< 0.001**^#^*
% filling therapy (n)	67.8 (437)	54.9 (180)	81.1 (257)	**< 0.001**^#^*
% professional teeth cleaning (n)	23.0 (149)	19.6 (65)	26.6 (84)	**0.034**^**#**^
Form of anesthesia				**< 0.001**^#^*
% GDA (n)	82.8 (540)	75.1 (251)	90.9 (289)	
% additional sedation	17.2 (112)	24.9 (83)	9.1 (29)	
Completion of therapy				**< 0.001**^#^*
% yes (n)	61.3 (399)	53.9 (180)	69.1 (219)	
% no (n)	12.9 (84)	13.8 (46)	12.0 (38)	
% unclear (n)	25.8 (168)	32.3 (108)	18.9 (60)	
% recall (n)	20.3 (132)	19.8 (66)	20.8 (66)	0.767^#^

Bold indicates significance level: *p* < 0.05

^#^Chi-Squared test

*Statistically significant

At the patient level, tooth extraction was considered first choice in both time periods, however more restorative approaches were taken between 2007 and 2011 including pulp therapy and the use of strip and stainless steel crowns compared to 2002–2006.

## Discussion

Among several oral disorders, dental caries is one of the most prevalent diseases in pediatric dental patients across the globe [25]. Especially early childhood caries is a global public health burden, in medical, social, and economic terms [26]. In the current study, a high caries experience was found in children aged 1–6 years undergoing GA with age and migration status being detected as relevant risk factors. National data from Germany for the years 2000–2009 show that the average dmft for 6-year-olds was 2.21 and 1.87, respectively [27, 28]. Caries experience in our study sample was found to be approximately five times higher with approximately 10 affected teeth. This is in agreement with other ECC studies reporting 8–9 affected teeth [29, 30]. Considering the young age, GA is the preferable option in these cases. Within GA, it is possible to restore optimal oral health in a single visit and prevent any anxiety.

Choice of dental treatment modality was found to be different in both time periods. In general, ECC treatment approaches under GA fall under two main categories: extractions only, or an approach that combines all treatments, including preventive, restorative and surgical procedures [31]. The choice is influenced by many factors: the restorability of the tooth, caries risk, parents' compliance and wishes, socioeconomical status, as well as the resources that are available [31]. In our study, significantly more patients were treated with restorative treatment approaches including strip crowns, stainless steel crowns, pulpotomies and fillings between 2007–2011 compared to 2002–2006. This might be explained by the resources available in the study center. In the second time period, dentists certified in pediatric dentistry were present with restorative procedures including endodontic treatment and crowns being routinely performed. Especially trends in pulp therapy were found to be different in our study. In the first time period (2002–2007) no tooth received pulpotomy and one teeth pulpectomy with calcium hydroxide as an intracanal medicament for disinfection. It can be assumed that these teeth were extracted as probably a broader approach was taken in these years. In the second time period (2007–2011) 66 teeth received pulpotomy and three teeth an endodontic treatment as described above. Moreover, much more SSC were placed between 2007 and 2011. This increase in using SSC is in accordance other retrospective studies. Chen et al. [21] observed an increasing trend in Taiwan towards the use of SSCs under GA over 10 years (2006–2015). However, they also showed that more primary teeth were extracted than endodontically treated. They argued that their children aged up to 6 years had rampant caries that involved the upper anterior primary teeth and an advanced caries extension, and in such circumstances, the tooth structure was seriously damaged. This is in accordance to the results of the present study; accordingly, extractions were still the most chosen option in this cohort at both time periods. However, the favorited approach in the study center was restoration of anterior teeth with strip crowns if possible, especially in later years.

Despite contemporary preventive and minimally invasive treatment approaches in pediatric dentistry, tooth extraction is still a necessary treatment for advanced and multisurface caries and the frequent choice of this treatment option shows again that ECC is progressing rapidly and children in need for GA often show severely damaged tooth structure.

Caries experience was found to be affected by age and migration background. However, these factors explain only 11.5% of variance in dmf-s. Furthermore, it should be taken into account that the majority of patients are male (almost 60%). Overall, within the present study the influencing factors were considered within a cohort of children with increased dental treatment demand who can be considered as a high-risk group. Socioeconomic factors at the community and family level, such as ethnicity are well-recognized determinants of oral health and identified as risk factors in several studies reviewed [32–34]. Parental education and employment status are also known to influence caries development [35].

Interestingly, caries experience was not affected by nutritional status in the current study. Overweight children did not have higher mean dmf-s scores compared to normal or underweight children. Thus, our hypothesis needed to be rejected. However, measuring the link between weight and pediatric dental health has always been controversial in the literature. A recent systematic review failed to draw clear conclusions [36]. Some studies showed that children with normal weight had significantly less caries in their primary teeth than the overweight children [37, 38]. In contrast, Chen et al. (1998) [39] did not find an association between dental caries in primary teeth and BMI in 3-year-old children. This was supported by recent studies [40, 41]. To date, the hypothesized nature of protective effect of the weight on dental caries experience in the primary dentition is still uncertain, as many other factors which influence this relationship have not been considered [38].

One limitation of our study regarding treatment option is that it was not possible to analyze if teeth that have been extracted in the first time period between 2002 and 2006 might have been suitable for pulp therapy. No pulpotomy was performed during these years. Furthermore, there might be a bias due to the fact that different operators were involved in the treatment under GA. However, certain strengths of our study can also be pointed out. These include the size of the cohort, the length of observation time over 10 years with a detailed focus on two time periods, as well as comprehensive data preparation and observation taking into consideration the age group and treatment under GA.

## Conclusions

This study highlights that children aged 1–6 years treated under GA in Germany show a high caries experience with age and migration background being identified as risk factors. Nutritional status showed no influence on caries experience. Regarding treatment approaches, tooth extractions were the most chosen treatment approach in both time periods due to advanced caries extension, however more restorative approaches were taken between 2007 and 2011 including pulp therapy and the use of strip and stainless steel crowns.

## Data Availability

The data that support the findings of this study are available from [DZ] but restrictions apply to the availability of these data, which were used under license for the current study, and so are not publicly available. Data are however available from the authors upon reasonable request and with permission of [DZ].

## Abbreviations

BMI: Body mass index
dmf-t/s: Decayed (d), missing (m), and filled (f) teeth (t)/surface (s) index
